# Comparative Evaluation of Polysaccharide Binders on the Quality Characteristics of Plant-Based Patties

**DOI:** 10.3390/foods12203731

**Published:** 2023-10-11

**Authors:** Jong-Hyeon Han, Dong-Hyun Keum, Seong-Joon Hong, Yea-Ji Kim, Sung-Gu Han

**Affiliations:** Department of Food Science and Biotechnology of Animal Resources, Konkuk University, Seoul 05029, Republic of Korea; hyeon4970@konkuk.ac.kr (J.-H.H.); illus4862@konkuk.ac.kr (D.-H.K.); rlfkrofkd@konkuk.ac.kr (S.-J.H.); dpwl961113@konkuk.ac.kr (Y.-J.K.)

**Keywords:** meat analog, plant-based patty, polysaccharide, texture, binder, organoleptic attribute

## Abstract

Polysaccharides have been used in the production of plant-based meat analogs to replicate the texture of real meat. However, there has been no study that comprehensively compares the effects of different polysaccharides, and a limited number of polysaccharides have been evaluated. Thus, we aimed to identify the most suitable polysaccharide and concentration for plant-based patties. Plant-based patties were manufactured by blending different concentrations (0%, 1%, and 2%) of six polysaccharides with other ingredients, and the quality characteristics and sensory properties were evaluated. The L* values of plant-based patties reduced during the cooking process resembled the color change of beef patty (BP). In particular, a 2% κ-carrageenan-added patty (Car-2) exhibited the lowest L* value among the plant-based patties, measured at 44.05 (*p* < 0.05). Texture parameters exhibited high values by adding 2% κ-carrageenan and locust bean gum, which was close to BP. In the sensory evaluation, Car-2 showed higher scores for sensory preferences than other plant-based patties. Based on our data, incorporating 2% κ-carrageenan could offer a feasible way of crafting plant-based meat analogs due to its potential to enhance texture and flavor. Further studies are required to evaluate the suitability of polysaccharides in various types of plant-based meat analogs.

## 1. Introduction

Meat products are widely consumed worldwide due to their nutrition, taste, flavor, and affordability. By 2020, global meat consumption amounted to approximately 330 million tons and is estimated to increase to 370 million tons by 2030 [1,2]. However, the increase in meat production is an unsustainable process that can lead to environmental problems, such as water depletion, climate change, deforestation, pollution, damage to hydrological and geological reserves, and loss of biodiversity [3]. Furthermore, the sustainability of meat as a primary food source is also a concern with respect to animal welfare and human health problems including cardiovascular diseases, cancers, and type 2 diabetes [4]. Therefore, there is a demand for meat analog that not only alleviates the concerns associated with meat consumption but also mimics the appearance, texture, taste, and flavor of meat.

Plant-based meat alternatives are a conventional and readily available type of meat analog made from plant protein, polysaccharides, water, and oil [5]. Incorporating legumes, whole grains, and vegetables into plant-based meat analogs has been reported to reduce the risk of chronic diseases and enhance overall health outcomes [6]. Furthermore, dietary fibers like methylcellulose and guar gum, utilized in plant-based meat analogs, have exhibited the ability to lower cholesterol and regulate glucose levels [7]. Plant proteins have a great capacity for water and oil absorption, and these properties facilitate the utilization of plant protein in complex food systems. The structuring process is necessary to achieve the desired meat-like texture. Textured vegetable protein (TVP) is a structured plant-based protein made through an extrusion process to mimic the fibrous structure of meat [8]. With TVP, polysaccharides and non-textured proteins can be used as a binder to enhance the water-holding capacity (WHC) and texture properties of various emulsion-based plant-based meat analogs [9]. Particularly, polysaccharides are a suitable binder to form a stable gel without a distinct color, taste, or odor. According to the type of polysaccharides used, a diverse structure can be formed, and it can affect the texture profile of foods [10]. For instance, various polysaccharides have been supplemented into meat products at various concentrations (0.5–2%) to improve their texture and serve as fat substitutes [11,12,13]. In addition, previous studies have investigated the effects of polysaccharides and their concentrations on plant-based meat alternatives [14,15]. Nonetheless, for plant-based meat analogs, there is a scarcity of research that concurrently evaluates the impacts of different polysaccharides. Also, compared to meat products, plant-based meat analogs have seen a more limited exploration of various polysaccharide types [16,17]. Therefore, a comparison study is necessary to explore the effects of polysaccharides in plant-based meat alternatives.

In the current study, we investigated the effects of various polysaccharides (e.g., κ-carrageenan, locust bean gum, arabic gum, gellan gum, guar gum, and xanthan gum) and their different concentrations (1% and 2%) on the physicochemical characteristics and sensory properties of plant-based patties. In addition, the most suitable polysaccharide and concentration that enhanced the overall quality of plant-based patties and closely resembled the characteristics of beef patties was selected.

## 2. Materials and Methods

### 2.1. Materials

Textured pea protein (TPP) was purchased from Sotexpro Co. (Paris, France). Polysaccharides (κ-carrageenan, locust bean gum, arabic gum, gellan gum, guar gum, and xanthan gum), salt, beet powder, and black pepper were purchased from ESfood Co. (Gyeonggi, Republic of Korea). Ginger powder and garlic powder were purchased from Oherb (Seoul, Republic of Korea). Konjac glucomannan and potato starch were purchased from Yujin Co. (Gyeonggi, Republic of Korea) and Daehan Flour Mills Co. (Seoul, Republic of Korea), respectively. Isolated pea protein (IPP) and yeast extract were purchased from Hyangrim Co. (Seoul, Republic of Korea) and Angel Yeast Co. (Hubei, China), respectively. Methylcellulose was purchased from LOTTE Fine Chemical Co. (Ulsan, Republic of Korea). Canola oil and coconut oil were purchased from CJ Cheiljedang (Seoul, Republic of Korea) and Palmtop Vegeoil Products Sdn. Bhd. (Johor, Malaysia), respectively. Beef patty (BP) was purchased from France Gourmet Co. (Gyeonggi, Republic of Korea).

### 2.2. Preparation of Plant-Based Patties

To evaluate the characteristics of plant-based patties supplemented with various polysaccharides, 14 formulations of patties were prepared based on the variety and concentrations of polysaccharides (Table 1): without polysaccharides (Control); κ-carrageenan 1% (Car-1); κ-carrageenan 2% (Car-2); locust bean gum 1% (LBG-1); locust bean gum 2% (LBG-2); arabic gum 1% (AG-1); arabic gum 2% (AG-2); gellan gum 1% (GeG-1); gellan gum 2% (GeG-2); guar gum 1% (GuG-1); guar gum 2% (GuG-2); xanthan gum 1% (XG-1); and xanthan gum 2% (XG-2). The ingredients in our formulation were selected based on previous studies [14,18,19]. To achieve the desired fundamental texture and taste of the plant-based patty, preliminary experiments such as color analyses, texture profile analyses (TPA), and sensory evaluations were conducted to determine the optimal concentrations of ingredients, such as TPP, methylcellulose, and various spices. To manufacture the plant-based patties, TPP was hydrated for 1 h at 4 °C and mixed with the ingredients containing different concentrations of polysaccharides for 6 min. A total of 110 g of the mixture was shaped into patties using a patty presser (manual burger press 4″, Spikomat Ltd., Nottingham, UK). BP served as the positive control group in the assessments of color, pH, and TPA and sensory evaluations. Patties were cooked using an electric pan (DW-1530, Daewon Home Electric Co., Ltd., Gyeonggi, Republic of Korea) at 150 °C for 3 min per side until the internal temperature of the patties reached 80 °C. The cooked patties were immediately used for sensory evaluation or allowed to cool to room temperature for 30 min before measuring physicochemical properties. All experiments were repeated three times. Within each replicate, four patties were made for each formulation.

### 2.3. Proximate Analysis

The moisture, protein, fat, and ash contents of plant-based patties before cooking were determined based on the Association of Official Analytical Chemists’ (AOAC) methods [20]. To measure the moisture content, samples were placed in an aluminum dish and dried in an oven (SW-90 D, Sang Woo Scientific Co., Bucheon, Republic of Korea) at 105 °C for 16 h. Protein content was measured using the Automatic Kjeldahl Nitrogen analyzer (Kjeltec Auto 2300 System II, Foss tecator AB, Hoganas, Sweden). To measure the fat content, samples were extracted based on the Soxhlet method using a solvent extraction system (Soxtec Avanti 2050 Auto System, Foss Tecator AB, Hoganas, Sweden). The ash content was measured by burning samples at 550 °C for 6 h in a muffle furnace. Total carbohydrates were calculated by subtracting the percentages of moisture, protein, fat, and ash contents from 100%. Each sample was measured three times.

### 2.4. Color and pH Measurement

The color of the patties’ surface was measured before and after cooking using a colorimeter (CR-210, Konica Minolta, Ltd., Osaka, Japan). The calibration of the colorimeter was adjusted using a white plate (L* = +97.27, a* = +5.21, b* = −3.40) and the color value was presented as L* (lightness), a* (redness), or b* (yellowness). Each sample was measured eight times.

To measure the pH value, 5 g of the sample was inoculated in 20 mL of distilled water and homogenized at 10,000 rpm for 1 min using a homogenizer (HG-15 A, DAIHAN Scientific Co., Ltd., Gangwon, Republic of Korea). Then, the pH value of homogenized samples was measured before and after cooking using a pH meter (LAQUA, Horiba, Kyoto, Japan) and each sample was measured eight times.

### 2.5. Water Holding Capacity and Cooking Yield of Plant-Based Patties

The WHC of plant-based patties was determined following the previously described method [10]. Ten grams of the raw patty were poured into a 50 mL conical tube and centrifuged at 6000× *g* for 15 min at 10 °C using a centrifuge (1580 MGR, GYROZEN, Gyeonggi, Republic of Korea). The samples were heated in a water bath for 15 min at 85 °C and cooled to room temperature. The samples were centrifuged once more at 6000× *g* for 15 min at 10 °C and the water was removed using a pipette. Each sample was measured three times and the WHC was calculated using the following equation:

WHC (%) = [1 − ((M + M1 − M2)/water content in the patty)] × 100

M = weight of the empty tube (g)

M1 = weight of the patty and tube (g)

M2 = weight of the patty and tube after heating and centrifugation (g)

The cooking yield of plant-based patties was determined by measuring the weight difference between raw and cooked patties. The cooked patty was cooled to room temperature for 30 min. Each sample was measured three times and the percentage of cooking yield was calculated using the following equation:

Cooking yield (%) = [weight of a cooked patty/weight of a raw patty] × 100

### 2.6. Texture Profile Analysis (TPA)

TPA of patties was performed using a texture analyzer (TA-XT plus, Stable Micro Systems Ltd., Surrey, Goldaming, UK) equipped with a 40 mm cylinder probe (P/40). Patties were cut into 1.5 × 1.5 × 1 cm and texture parameters (hardness, springiness, cohesiveness, chewiness, and gumminess) were measured under the following conditions: pre-test speed 2.0 mm/s; test speed 1.0 mm/s; post-test speed 1.0 mm/s; force 5 g. Each sample was measured eight times.

### 2.7. Sensory Evaluation

The sensory evaluation of patties was conducted following the previously described method with slight modifications [21]. Ten trained panelists (six males and four females aged from 24 to 30 who were students at the Konkuk University Food Science Department) were recruited for the sensory evaluation of patties. The training consisted of introducing the patties in initial sessions to acquaint the panelists with the characteristics to be assessed. The panelists underwent training using identical testing samples in three training sessions lasting 20–30 min each for 2 weeks before the formal sensory evaluations. During formal analysis, the samples were cut into 1.5 × 1.5 × 1 cm and a 3-digit random number was assigned to each sample. After one sample was evaluated, the panelists were instructed to cleanse their palates using water. Subsequently, samples were evaluated using a 9-point scale to assess their juiciness intensity (9 = juicy; 1 = dry), firmness intensity (9 = extremely firm; 1 = extremely soft), appearance, taste, flavor, firmness preference, and overall acceptability (9 = extremely like; 1 = extremely dislike). The Institutional Review Board approved the procedure of sensory evaluation (7001355-202303-HR-637).

### 2.8. Statistical Analysis

All the data are expressed as mean ± standard deviation. All analyses were performed using SPSS-PASW statistics software version 22.0 (SPSS Inc., Chicago, IL, USA). The statistical significance was analyzed using a one-way analysis of variance (ANOVA) with Duncan’s multiple range post hoc test to determine significant differences (*p* < 0.05) between the groups.

## 3. Results and Discussion

### 3.1. Proximate Analysis of Plant-Based Patties

Moisture, protein, fat, ash, and total carbohydrate contents of raw patties are shown in Table 2. The moisture content of the patties was significantly decreased by the addition of polysaccharides compared to the control (*p* < 0.05). It seemed to be directly affected by the reduced water content in the formulation. The similarity between the added water quantity and the actual moisture content of the products before heating suggests that the formulation of the patties was effective in retaining the added water. When the amounts of TVP and other binders were inadequate, water and oil could be separated from the raw patties. Conversely, the water retention capacity and gelling properties of polysaccharides were likely to exert a positive influence on the plant-based meat analog [9]. The protein content of plant-based patties ranged from 18.08% to 19.62%, which was comparable to that of beef patties [14]. Since meat analogs have received attention as an alternative protein source, ensuring that their protein content matches that of meat products is crucial [22]. Our formulation was optimal to offer protein levels comparable to those found in meat patties. The fat content of the patties ranged from 8.95% to 10.24%, which was comparable to that of commercial plant-based products and approximately half as low as that of ground beef patties [23]. Moreover, half of the oil incorporated into our formulation consisted of canola oil, primarily composed of unsaturated fatty acids (91.7%) [24]. One of the concerns related to meat consumption is the increased risk of disease due to its high saturated fat content [25]. Thus, our formulation looks optimal in providing less saturated fat content to alleviate the health concerns associated with meat consumption. The variation in ash content was also minor and within the range of market meat analog products. The addition of polysaccharides resulted in an overall increase in the total carbohydrate content. The overall range of total carbohydrate content was between 13.16% and 15.71%. Total carbohydrates included sugar, starch, and fiber, and the polysaccharides used in our study belonged to the category of soluble dietary fiber [26]. Thus, the addition of fiber-rich polysaccharides could contribute to the overall increase in total carbohydrate content. Overall, the addition of polysaccharides had an impact on the moisture, protein, fat, and ash contents of the plant-based patties, but the range of values did not appear to have a significant nutritional difference.

### 3.2. Color and pH of Plant-Based Patties

**Figure 1:**
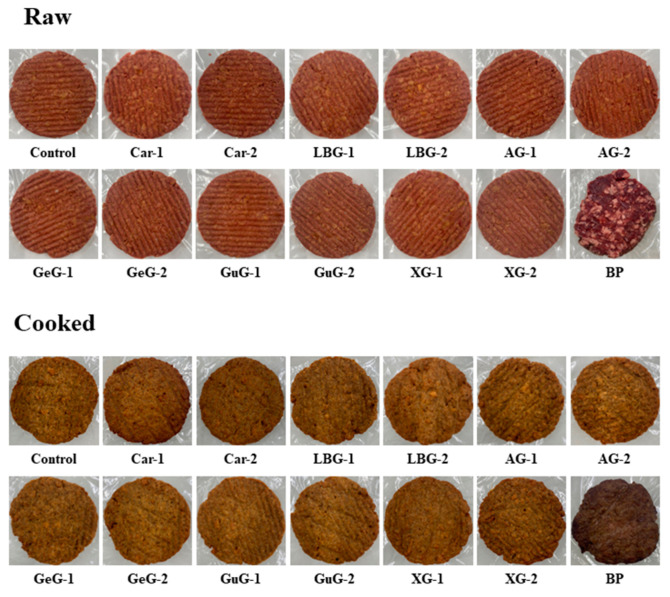
Visual appearance of plant-based patties supplemented with different concentrations of polysaccharides and beef patty. Control: plant-based patty without polysaccharide; Car-1: plant-based patty with 1% κ-carrageenan added; Car-2: plant-based patty with 2% κ-carrageenan added; LBG-1: plant-based patty with 1% locust bean gum added; LBG-2: plant-based patty with 2% locust bean gum added; AG-1: plant-based patty with 1% arabic gum added; AG-2: plant-based patty with 2% arabic gum added; GeG-1: plant-based patty with 1% gellan gum added; GeG-2: plant-based patty with 2% gellan gum added; GuG-1: plant-based patty with 1% guar gum added; GuG-2: plant-based patty with 2% guar gum added; XG-1: plant-based patty with 1% xanthan gum added; XG-2: plant-based patty with 2% xanthan gum added; BP: beef patty.

The color and pH of plant-based patties and BP are shown in Table 3 and Figure 1. The L* value of plant-based patties before cooking showed no significant difference and, likewise, there were no significant effects on the L* value of BP (*p* > 0.05). Meat color is an important factor for assessing quality, with the red color of uncooked meat having the most significant impact on consumers’ product preferences [27]. Although, the a* value of BP was 19.04, indicating a significant difference compared to the plant-based patty groups (*p* < 0.05), the a* value generally increased with the addition of polysaccharides. Especially among the plant-based patty groups, the a* value of AG-2 and Car-2 was similar to that of BP. The b* value of plant-based patties before cooking was significantly increased with the addition of κ-carrageenan, locust bean gum, and arabic gum (*p* < 0.05). Furthermore, the b* value of Car-1 and Car-2 was adjacent to that of BP (*p* > 0.05). The color of BP was changed during the cooking process, and all of the color coordinates and L*, a*, and b* values were generally decreased. Our data indicate that the L* value of plant-based patties after cooking tended to decrease with the supplementation of polysaccharides. Specifically, the L* value of the plant-based patty in Car-2 exhibited a lower value than the other plant-based patty groups (*p* < 0.05); however, it remained higher than that for the BP. Overall, the addition of polysaccharides did not significantly affect the a* value of cooked plant-based patties (*p* > 0.05). However, it showed a decreasing trend compared to the a* value of raw patties, which was like the change in raw and cooked BP. During the heating process, meat loses its red color at around 65 °C due to the denaturation of myoglobin [28]. In the current study, beet powder was added to provide the red color to the plant-based patties. However, during the heating process, betalain, a pigment found in beet, underwent denaturation and lost its red color, leading to a decrease in the a* value [29,30]. The b* value of plant-based patties after cooking showed no significant difference with the addition of polysaccharides (*p* > 0.05), although the b^*^ value of BP was significantly lower than that for the other plant-based patty groups. Therefore, the formulation employed in our study exhibited a color change trend like that of BP, with Car-2 being identified as the most effective.

The pH values of raw plant-based patties were in the range of 6.38 to 6.56 and that of cooked plant-based patties ranged between 6.25 and 6.37. In the case of BP, the pH value of raw patties was 5.52 and that of cooked patties was 5.71. Due to the glycolysis system after slaughter, the pH value of normal fresh meat is reduced from 7.2 to 5.5 [31,32]. An undesirable pH value of meat (above 6.2) can hinder the oxygen delivery of myoglobin or cause protein denaturation, leading to a deterioration in meat color [33]. Therefore, the pH value of meat is a critical factor that affects its quality. However, plant-based patties made with TPP did not undergo postmortem glycolysis and, due to their higher buffering capacity compared to meat patties, they experienced less pH variation [34]. Thus, the color of plant-based patties was more influenced by the type of coloring agents rather than pH changes, indicating that the pH difference in plant-based patties was relatively insignificant [35]. The pH value of the raw and cooked plant-based patties was lowest in XG-2 (*p* < 0.05). Consistent with our results, the pH value of mutton meatballs containing xanthan gum was decreased due to pyruvic acid and gluconic acid, which constitute the structure of xanthan gum [13]. However, the maximum difference in pH values between the control group and the polysaccharide-added groups was 0.11, which could be considered negligible [12]. Therefore, based on the formulation of the plant-based patties in this study, it could be concluded that the addition of polysaccharides had little impact on the pH.

### 3.3. Water Holding Capacity (WHC) and Cooking Yield of Plant-Based Patties

**Figure 2:**
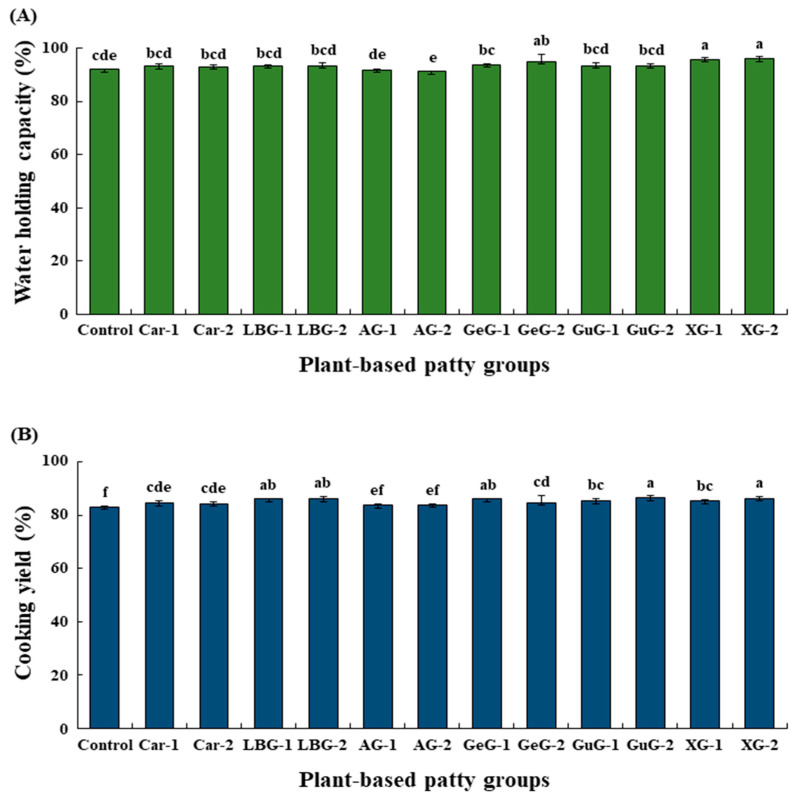
Water holding capacity (WHC) and cooking yield of plant-based patties supplemented with different concentrations of polysaccharides. (**A**) WHC and (**B**) cooking yield of plant-based patties. Control: plant-based patty without polysaccharide; Car-1: plant-based patty with 1% κ-carrageenan added; Car-2: plant-based patty with 2% κ-carrageenan added; LBG-1: plant-based patty with 1% locust bean gum added; LBG-2: plant-based patty with 2% locust bean gum added; AG-1: plant-based patty with 1% arabic gum added; AG-2: plant-based patty with 2% arabic gum added; GeG-1: plant-based patty with 1% gellan gum added; GeG-2: plant-based patty with 2% gellan gum added; GuG-1: plant-based patty with 1% guar gum added; GuG-2: plant-based patty with 2% guar gum added; XG-1: plant-based patty with 1% xanthan gum added; XG-2: plant-based patty with 2% xanthan gum added. The error bars indicate standard deviation (n = 3). ^a–f^ Different letters represent a significant difference (*p* < 0.05).

The WHC of plant-based patties is shown in Figure 2A. Except for groups that contained xanthan gum or gellan gum, the WHC of plant-based patties was not significantly different from the control in all samples (*p* > 0.05). The WHC was used to measure the ability of plant-based patties to retain water during the heating process. TPP was denatured into a porous structure by high temperature and high pressure during the extrusion process [36,37]. Furthermore, since a textured protein was already aggregated during extrusion, protein denaturation did not occur even at temperatures between 80–100 °C [8]. Therefore, TPP had excellent water absorption abilities due to its porous structure, and protein denaturation did not occur at the temperature of 85 °C. Similarly, our data also showed that the WHC of the control was 92.13%, indicating excellent water binding. While it was commonly accepted that polysaccharides enhance WHC, our finding indicated that the addition of polysaccharides to plant-based patties has no significant effect on their WHC due to the excellent water entrapment ability of TPP.

The cooking yield of plant-based patties is shown in Figure 2B. Except for the groups that contained arabic gum, the addition of polysaccharides significantly increased the cooking yield compared to the control (*p* < 0.05). The cooking yield measured the amount of moisture lost by comparing the weight of the patties before and after cooking. The addition of polysaccharides to the plant-based patties resulted in a slight increase in WHC, but it was not significant, possibly due to the stable structure at 85 °C and excellent water entrapment ability of TPP. However, our cooking yield results showed a significant increase with the addition of polysaccharides to plant-based patties. Although the internal temperature of the plant-based patty reached 80 °C during the cooking process, the surface portion was heated to 150 °C, which was similar to the TVP extrusion temperature [37]. This might cause the surface of the TVP to denature and fragment the protein structure, resulting in the release of moisture [38]. Then, the released moisture could be absorbed by the added polysaccharide, leading to a significant increase in the cooking yield of plant-based patties. In a previous study, a single addition of a binder did not significantly affect the WHC of plant-based patties but increased the cooking yield [21]. Therefore, our data suggested that polysaccharides prevent moisture release from the surface during the cooking process of plant-based patties.

### 3.4. Texture Profile Analysis of Plant-Based Patties

The TPA of plant-based patties and BP is shown in Table 4. The type and concentration of added polysaccharide influenced the texture parameters of patties. Car-2 and LBG-2 showed significantly higher values of texture parameters such as hardness, cohesiveness, chewiness, and gumminess than other plant-based patties (*p* < 0.05). In particular, all texture parameters of Car-2 were close to those of BP. A previous study reported that the incorporation of konjac-glucomannan and κ-carrageenan blends into soy-based meat analogs increased their tensile strength, breaking time, and hardness, resulting in outcomes comparable to those observed in beef samples [39]. Konjac-glucomannan is known to interact with polysaccharides, leading to increased gel strength, viscosity, and elasticity. Particularly, κ-carrageenan has been found to exhibit the most substantial synergistic effect among the polysaccharides [40]. In addition, adding 0.2% of κ-carrageenan to turkey meat sausage decreased compactness as it could not bind with the protein gel network, while a 1.5% concentration contributed to structured protein gel network formation and increased hardness, cohesiveness, and chewiness [41]. Thus, we thought that Car-2 created a balanced binding between moisture, pea protein, and konjac-glucomannan, thereby contributing to an increase in hardness, springiness, cohesiveness, chewiness, and gumminess.

XG-2 and AG-2 resulted in a significant decrease in hardness, springiness, chewiness, and gumminess compared to the control (*p* < 0.05). Xanthan gum formed a long, string-like structure that resulted in high hardness due to suitable interactions with water molecules and proteins when added at an appropriate concentration [42,43]. However, adding an excessive concentration of xanthan gum increased the ratio of hydrogen bonding with water, leading to increased water absorption but the weakened emulsion structures of proteins such as IPP, which resulted in a softer texture [42]. Hence, the decrease in the texture parameters of XG-2 was thought to be caused by an imbalanced binding among excessive concentrations of xanthan gum, water, and protein.

### 3.5. Sensory Evaluation of Plant-Based Patties

**Figure 3:**
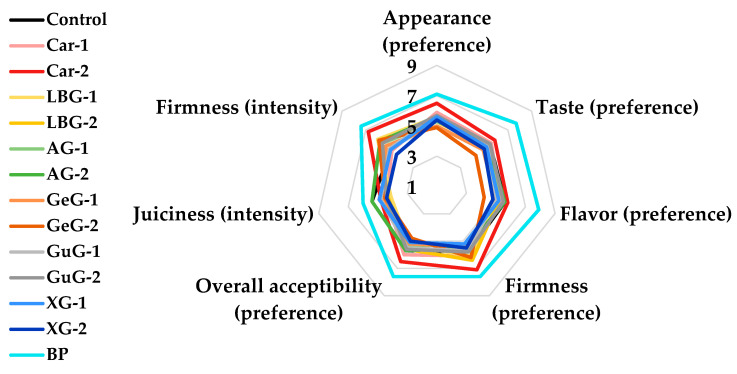
Sensory properties of plant-based patties supplemented with different concentrations of polysaccharides and beef patties. Each colored line indicates a different sample. Control: plant-based patty without polysaccharide; Car-1: plant-based patty with 1% κ-carrageenan added; Car-2: plant-based patty with 2% κ-carrageenan added; LBG-1: plant-based patty with 1% locust bean gum added; LBG-2: plant-based patty with 2% locust bean gum added; AG-1: plant-based patty with 1% arabic gum added; AG-2: plant-based patty with 2% arabic gum added; GeG-1: plant-based patty with 1% gellan gum added; GeG-2: plant-based patty with 2% gellan gum added; GuG-1: plant-based patty with 1% guar gum added; GuG-2: plant-based patty with 2% guar gum added; XG-1: plant-based patty with 1% xanthan gum added; XG-2: plant-based patty with 2% xanthan gum added; BP: beef patty. The experiment was conducted with ten panelists (n = 10).

Sensory evaluations regarding preference and intensity are often conducted with trained panelists to evaluate the inherent qualities of food and ingredients [21,44]. The mean scores of sensory categories of plant-based patties and BP are shown in Figure 3. The juiciness score was generally lower in all plant-based patty groups than for BP, while polysaccharide-added groups showed even lower scores compared to the control. This may be because the amount of water added to the formulation was reduced by incorporating polysaccharides, and thus the water content of the patties was decreased. Additionally, the impact of polysaccharides on enhancing WHC was found to be insignificant. Consistent with our hypothesis, the addition of a binder to pork sausage resulted in a decreased juiciness score, which could be attributed to the lower water content in the formulation compared to the control group [45]. In our next studies, investigating the use of polysaccharides in the form of hydrogels or oleogels could be a promising strategy to mitigate the reduction in juiciness that was observed in the current study [46,47]. In all sensory preference categories, BP received the highest scores within the group. However, the Car-2 group exhibited higher preference scores than the other plant-based patties, with notable score differences observed in appearance and firmness. The low L* value of Car-2 after cooking suggested that the patty had a darker brown color compared to the other plant-based patties (Table 3, Figure 1). Thus, it was assumed that the darker brown color gave Car-2 a meat-like appearance, resulting in a higher appearance score from the panelists. Furthermore, the high values of texture parameters in Car-2 were associated with high firmness intensity scores from the panelists (*p* < 0.05), indicating that the intensity level of firmness was satisfactory, according to the panel’s preferences. From the same perspective, there were no significant differences in the appearance and firmness scores of BP (*p* > 0.05) as they exhibited relatively similar color and texture properties to those of Car-2. Collectively, the addition of 2% of κ-carrageenan to plant-based patties could improve overall acceptability by improving appearance and firmness.

## 4. Conclusions

The effects of various polysaccharides and their concentrations on the physicochemical characteristics and sensory properties of plant-based patties were determined. The moisture and fat content were decreased, and the total carbohydrate content was increased by adding the polysaccharides. The color of plant-based patties was also affected by polysaccharides; in particular, the lightness of patties after cooking was decreased by the addition of 2% of κ-carrageenan (*p* < 0.05). The increase of WHC was insignificant, and cooking yield was slightly improved by polysaccharides. While the texture profiles of patties varied depending on the type and concentration of polysaccharides, the addition of 2% of κ-carrageenan highly increased the texture parameters, which were the most closely adjacent to those of BP. Appearance and firmness were improved and overall acceptability was the highest in the 2% κ-carrageenan-added patty. Based on our data, it appears that incorporating polysaccharides into plant-based patties can enhance their physicochemical properties overall. In particular, 2% of κ-carrageenan appeared to be the most effective ingredient for replicating the appearance and texture of a beef patty. Based on our findings, incorporating 2% of κ-carrageenan appears to be a promising approach for creating a high-quality meat patty alternative. Our data could enhance both appearance and texture, which are critical factors influencing consumer preferences both before and during consumption. However, our data are limited to application in plant-based patty products employing textured pea protein and the specific ingredients used in our study. Consequently, future research is necessary with varying concentrations and types of polysaccharides in different types of plant-based meat analogs, utilizing alternative textured vegetable proteins as well.

## Figures and Tables

**Table 1:** Formulation percentage of plant-based patties supplemented with different concentrations of polysaccharides.

Ingredients (%)/Patty Groups	Control	Car-1	Car-2	LBG-1	LBG-2	AG-1	AG-2	GeG-1	GeG-2	GuG-1	GuG-2	XG-1	XG-2	BP
Textured pea protein	20	20	20	20	20	20	20	20	20	20	20	20	20	0
Water	56.0725	55.0725	54.0725	55.0725	54.0725	55.0725	54.0725	55.0725	54.0725	55.0725	54.0725	55.0725	54.0725	0
Methylcellulose	2	2	2	2	2	2	2	2	2	2	2	2	2	0
Konjac glucomannan	0.5	0.5	0.5	0.5	0.5	0.5	0.5	0.5	0.5	0.5	0.5	0.5	0.5	0
κ-Carrageenan	0	1	2	0	0	0	0	0	0	0	0	0	0	0
Locust bean gum	0	0	0	1	2	0	0	0	0	0	0	0	0	0
Arabic gum	0	0	0	0	0	1	2	0	0	0	0	0	0	0
Gellan gum	0	0	0	0	0	0	0	1	2	0	0	0	0	0
Guar gum	0	0	0	0	0	0	0	0	0	1	2	0	0	0
Xanthan gum	0	0	0	0	0	0	0	0	0	0	0	1	2	0
Salt	1.1	1.1	1.1	1.1	1.1	1.1	1.1	1.1	1.1	1.1	1.1	1.1	1.1	0
Beet powder	0.75	0.75	0.75	0.75	0.75	0.75	0.75	0.75	0.75	0.75	0.75	0.75	0.75	0
Ginger powder	0.3275	0.3275	0.3275	0.3275	0.3275	0.3275	0.3275	0.3275	0.3275	0.3275	0.3275	0.3275	0.3275	0
Isolated pea protein	4.75	4.75	4.75	4.75	4.75	4.75	4.75	4.75	4.75	4.75	4.75	4.75	4.75	0
Garlic powder	1	1	1	1	1	1	1	1	1	1	1	1	1	0
Yeast extract	1	1	1	1	1	1	1	1	1	1	1	1	1	0
Black pepper	0.5	0.5	0.5	0.5	0.5	0.5	0.5	0.5	0.5	0.5	0.5	0.5	0.5	0
Canola oil	6	6	6	6	6	6	6	6	6	6	6	6	6	0
Coconut oil	6	6	6	6	6	6	6	6	6	6	6	6	6	0
Lean beef	0	0	0	0	0	0	0	0	0	0	0	0	0	80
Beef back fat	0	0	0	0	0	0	0	0	0	0	0	0	0	20
Total	100	100	100	100	100	100	100	100	100	100	100	100	100	100

Control: plant-based patty without polysaccharide; Car-1: plant-based patty with 1% κ-carrageenan added; Car-2: plant-based patty with 2% κ-carrageenan added; LBG-1: plant-based patty with 1% locust bean gum added; LBG-2: plant-based patty with 2% locust bean gum added; AG-1: plant-based patty with 1% arabic gum added; AG-2: plant-based patty with 2% arabic gum added; GeG-1: plant-based patty with 1% gellan gum added; GeG-2: plant-based patty with 2% gellan gum added; GuG-1: plant-based patty with 1% guar gum added; GuG-2: plant-based patty with 2% guar gum added; XG-1: plant-based patty with 1% xanthan gum added; XG-2: plant-based patty with 2% xanthan gum added; BP: beef patty.

**Table 2:** Proximate analysis of plant-based patties supplemented with different concentrations of polysaccharides.

Parameters	Plant-Based Patty Groups												
	Control	Car-1	Car-2	LBG-1	LBG-2	AG-1	AG-2	GeG-1	GeG-2	GuG-1	GuG-2	XG-1	XG-2
Moisture (%)	56.37 ± 0.31 ^a^	55.68 ± 0.15 ^cd^	54.41 ± 0.05 ^f^	55.85 ± 0.12 ^bc^	54.72 ± 0.21 ^e^	56.10 ± 0.17 ^ab^	54.72 ± 0.17 ^e^	55.69 ± 0.28 ^cd^	54.84 ± 0.21 ^e^	55.78 ± 0.09 ^bc^	54.98 ± 0.19 ^e^	55.39 ± 0.05 ^d^	54.39 ± 0.11 ^f^
Protein (%)	18.08 ± 0.18 ^d^	18.53 ± 0.05 ^bc^	18.29 ± 0.19 ^cd^	18.67 ± 0.02 ^b^	18.16 ± 0.06 ^d^	18.69 ± 0.17 ^b^	18.36 ± 0.18 ^cd^	18.17 ± 0.09 ^d^	19.62 ± 0.05 ^a^	18.59 ± 0.19 ^bc^	18.59 ± 0.25 ^bc^	18.33 ± 0.11 ^cd^	18.36 ± 0.27 ^cd^
Fat (%)	10.20 ± 0.23 ^a^	9.38 ± 0.06 ^c^	9.72 ± 0.08 ^b^	9.79 ± 0.14 ^b^	9.59 ± 0.06 ^bc^	9.35 ± 0.12 ^c^	8.95 ± 0.08 ^d^	9.67 ± 0.14 ^b^	9.76 ± 0.10 ^b^	10.24 ± 0.29 ^a^	9.58 ± 0.13 ^bc^	9.76 ± 0.18 ^b^	9.72 ± 0.11 ^b^
Ash (%)	2.19 ± 0.06 ^ef^	2.31 ± 0.07 ^bc^	2.45 ± 0.04 ^a^	2.21 ± 0.03 ^def^	2.27 ± 0.01 ^cde^	2.25 ± 0.03 ^cdef^	2.25 ± 0.02 ^cdef^	2.37 ± 0.06 ^b^	2.47 ± 0.06 ^a^	2.19 ± 0.01 ^ef^	2.18 ± 0.03 ^f^	2.29 ± 0.06 ^cd^	2.30 ± 0.05 ^bc^
Total Carbohydrates (%)	13.16 ± 0.34 ^g^	14.10 ± 0.24 ^ef^	15.13 ± 0.21 ^bc^	13.48 ± 0.10 ^g^	15.27 ± 0.14 ^ab^	13.61 ± 0.16 ^fg^	15.71 ± 0.30 ^a^	14.10 ± 0.20 ^ef^	13.31 ± 0.29 ^g^	13.19 ± 0.39 ^g^	14.68 ± 0.52 ^cd^	14.23 ± 0.21 ^de^	15.24 ± 0.28 ^ab^

Control: plant-based patty without polysaccharide; Car-1: plant-based patty with 1% κ-carrageenan added; Car-2: plant-based patty with 2% κ-carrageenan added; LBG-1: plant-based patty with 1% locust bean gum added; LBG-2: plant-based patty with 2% locust bean gum added; AG-1: plant-based patty with 1% arabic gum added; AG-2: plant-based patty with 2% arabic gum added; GeG-1: plant-based patty with 1% gellan gum added; GeG-2: plant-based patty with 2% gellan gum added; GuG-1: plant-based patty with 1% guar gum added; GuG-2: plant-based patty with 2% guar gum added; XG-1: plant-based patty with 1% xanthan gum added; XG-2: plant-based patty with 2% xanthan gum added. Data are presented as mean ± standard deviation (n = 3). ^a–g^ Different letters in a row represent a significant difference (*p* < 0.05).

**Table 3:** Color and pH of plant-based patties supplemented with different concentrations of polysaccharides and beef patty.

Parameters		Plant-Based Patty Groups													
		Control	Car-1	Car-2	LBG-1	LBG-2	AG-1	AG-2	GeG-1	GeG-2	GuG-1	GuG-2	XG-1	XG-2	BP
Raw	L*	51.22 ± 0.17 ^ab^	50.92 ± 0.66 ^ab^	50.96 ± 0.67 ^ab^	51.02 ± 0.53 ^ab^	51.00 ± 0.65 ^ab^	50.64 ± 0.35 ^b^	50.66 ± 0.36 ^b^	50.95 ± 0.64 ^ab^	51.13 ± 0.49 ^ab^	50.64 ± 0.23 ^b^	50.68 ± 0.65 ^b^	51.57 ± 0.40 ^a^	51.52 ± 0.57 ^a^	51.01 ± 0.58 ^ab^
	a*	16.30 ± 0.19 ^gh^	17.50 ± 0.41 ^d^	17.59 ± 0.17 ^cd^	16.80 ± 0.38 ^ef^	16.94 ± 0.42 ^e^	17.92 ± 0.27 ^bc^	18.08 ± 0.37 ^b^	16.22 ± 0.27 ^h^	16.46 ± 0.28 ^fgh^	16.20 ± 0.17 ^h^	16.26 ± 0.24 ^gh^	16.66 ± 0.38 ^efg^	17.02 ± 0.34 ^e^	19.04 ± 0.70 ^a^
	b*	13.02 ± 0.14 ^de^	13.90 ± 0.23 ^ab^	13.95 ± 0.12 ^ab^	13.24 ± 0.15 ^cd^	13.38 ± 0.21 ^c^	13.73 ± 0.21 ^b^	14.11 ± 0.16 ^a^	13.21 ± 0.09 ^cd^	13.28 ± 0.48 ^cd^	13.25 ± 0.12 ^cd^	13.16 ± 0.31 ^cd^	12.74 ± 0.25 ^e^	12.72 ± 0.22 ^e^	13.85 ± 0.57 ^ab^
	pH	6.49 ± 0.03 ^cd^	6.51 ± 0.01 ^bc^	6.52 ± 0.04 ^bc^	6.50 ± 0.02 ^bcd^	6.54 ± 0.01 ^ab^	6.49 ± 0.02 ^cd^	6.42 ± 0.01 ^f^	6.50 ± 0.02 ^bcd^	6.46 ± 0.00 ^de^	6.51 ± 0.01 ^bc^	6.56 ± 0.01 ^a^	6.43 ± 0.01 ^ef^	6.38 ± 0.05 ^g^	5.52 ± 0.05 ^h^
Cooked	L*	47.60 ± 1.31 ^a^	44.73 ± 0.58 ^cd^	44.05 ± 0.94 ^d^	45.68 ± 0.95 ^bc^	45.41 ± 0.94 ^c^	45.33 ± 0.58 ^c^	45.22 ± 0.48 ^c^	46.68 ± 0.91 ^ab^	46.59 ± 0.94 ^ab^	45.78 ± 1.06 ^bc^	45.50 ± 0.72 ^c^	46.96 ± 0.45 ^ab^	46.97 ± 1.37 ^ab^	41.11 ± 1.26 ^e^
	a*	7.23 ± 0.29 ^a^	7.22 ± 0.49 ^a^	7.30 ± 0.52 ^a^	7.27 ± 0.37 ^a^	7.29 ± 0.29 ^a^	7.23 ± 0.19 ^a^	7.28 ± 0.29 ^a^	7.11 ± 0.35 ^a^	7.35 ± 0.17 ^a^	7.20 ± 0.27 ^a^	7.19 ± 0.13 ^a^	7.23 ± 0.35 ^a^	7.35 ± 0.41 ^a^	5.50 ± 0.30 ^b^
	b*	17.48 ± 0.54 ^a^	17.37 ± 0.35 ^a^	17.61 ± 0.61 ^a^	17.36 ± 0.93 ^a^	17.39 ± 0.34 ^a^	17.47 ± 0.23 ^a^	17.34 ± 0.75 ^a^	17.16 ± 0.72 ^a^	17.61 ± 0.28 ^a^	17.47 ± 0.66 ^a^	17.58 ± 0.30 ^a^	17.58 ± 0.73 ^a^	17.57 ± 1.21 ^a^	5.80 ± 0.61 ^b^
	pH	6.36 ± 0.01 ^ab^	6.35 ± 0.01 ^ab^	6.30 ± 0.00 ^d^	6.37 ± 0.01 ^a^	6.37 ± 0.01 ^a^	6.35 ± 0.02 ^ab^	6.29 ± 0.02 ^d^	6.36 ± 0.01 ^ab^	6.37 ± 0.04 ^a^	6.33 ± 0.01 ^bc^	6.37 ± 0.01 ^a^	6.31 ± 0.00 ^cd^	6.25 ± 0.01 ^e^	5.71 ± 0.01 ^f^

Control: plant-based patty without polysaccharide; Car-1: plant-based patty with 1% κ-carrageenan added; Car-2: plant-based patty with 2% κ-carrageenan added; LBG-1: plant-based patty with 1% locust bean gum added; LBG-2: plant-based patty with 2% locust bean gum added; AG-1: plant-based patty with 1% arabic gum added; AG-2: plant-based patty with 2% arabic gum added; GeG-1: plant-based patty with 1% gellan gum added; GeG-2: plant-based patty with 2% gellan gum added; GuG-1: plant-based patty with 1% guar gum added; GuG-2: plant-based patty with 2% guar gum added; XG-1: plant-based patty with 1% xanthan gum added; XG-2: plant-based patty with 2% xanthan gum added; BP: beef patty. Data are presented as mean ± standard deviation (n = 8). ^a–h^ Different letters in a row represent a significant difference (*p* < 0.05).

**Table 4:** Texture profile analysis of plant-based patties supplemented with different concentrations of polysaccharides and beef patty.

Parameters	Plant-Based Patty Groups													
	Control	Car-1	Car-2	LBG-1	LBG-2	AG-1	AG-2	GeG-1	GeG-2	GuG-1	GuG-2	XG-1	XG-2	BP
Hardness (N)	8.88 ± 0.78 ^e^	11.37 ± 0.97 ^bc^	14.90 ± 1.12 ^a^	9.29 ± 0.97 ^de^	12.33 ± 1.34 ^b^	6.67 ± 0.85 ^fg^	6.06 ± 0.55 ^g^	8.82 ± 0.75 ^e^	10.56 ± 1.18 ^cd^	9.03 ± 0.94 ^e^	9.36 ± 1.28 ^de^	7.44 ± 1.53 ^f^	7.12 ± 0.89 ^fg^	15.28 ± 1.94 ^a^
Springiness	0.48 ± 0.02 ^f^	0.57 ± 0.01 ^c^	0.67 ± 0.01 ^b^	0.50 ± 0.01 ^e^	0.55 ± 0.03 ^d^	0.46 ± 0.03 ^g^	0.45 ± 0.03 ^g^	0.49 ± 0.01 ^ef^	0.54 ± 0.01 ^d^	0.39 ± 0.01 ^h^	0.39 ± 0.01 ^h^	0.44 ± 0.01 ^g^	0.39 ± 0.01 ^h^	0.82 ± 0.03 ^a^
Cohesiveness	0.38 ± 0.01 ^de^	0.39 ± 0.01 ^c^	0.44 ± 0.01 ^b^	0.40 ± 0.02 ^c^	0.43 ± 0.01 ^b^	0.37 ± 0.02 ^e^	0.36 ± 0.02 ^e^	0.38 ± 0.02 ^de^	0.39 ± 0.02 ^cd^	0.32 ± 0.01 ^f^	0.32 ± 0.01 ^f^	0.37 ± 0.01 ^e^	0.36 ± 0.02 ^e^	0.69 ± 0.02 ^a^
Chewiness (N)	1.46 ± 0.23 ^ef^	2.37 ± 0.16 ^d^	3.92 ± 0.32 ^b^	1.69 ± 0.08 ^e^	2.78 ± 0.23 ^c^	1.08 ± 0.18 ^g^	0.97 ± 0.27 ^g^	1.48 ± 0.14 ^ef^	2.19 ± 0.16 ^d^	1.23 ± 0.16 ^fg^	1.27 ± 0.19 ^fg^	1.33 ± 0.25 ^efg^	1.02 ± 0.11 ^g^	7.10 ± 0.91 ^a^
Gumminess (N)	3.43 ± 0.30 ^efg^	4.40 ± 0.22 ^d^	6.27 ± 0.59 ^b^	3.72 ± 0.38 ^ef^	5.74 ± 0.60 ^c^	2.38 ± 0.28 ^h^	2.20 ± 0.42 ^h^	3.35 ± 0.41 ^fg^	3.87 ± 0.39 ^e^	3.08 ± 0.35 ^g^	3.10 ± 0.26 ^g^	2.42 ± 0.43 ^h^	2.34 ± 0.31 ^h^	9.81 ± 0.79 ^a^

Control: plant-based patty without polysaccharide; Car-1: plant-based patty with 1% κ-carrageenan added; Car-2: plant-based patty with 2% κ-carrageenan added; LBG-1: plant-based patty with 1% locust bean gum added; LBG-2: plant-based patty with 2% locust bean gum added; AG-1: plant-based patty with 1% arabic gum added; AG-2: plant-based patty with 2% arabic gum added; GeG-1: plant-based patty with 1% gellan gum added; GeG-2: plant-based patty with 2% gellan gum added; GuG-1: plant-based patty with 1% guar gum added; GuG-2: plant-based patty with 2% guar gum added; XG-1: plant-based patty with 1% xanthan gum added; XG-2: plant-based patty with 2% xanthan gum added; BP: beef patty. Data are presented as mean ± standard deviation (n = 8). ^a–h^ Different letters in a row represent a significant difference (*p* < 0.05).

## Data Availability

The data used to support the findings of this study can be made available by the corresponding author upon request.
